# Synovial Fluid Inflammatory Profiles did not Differ between Isolated Anterior Cruciate Ligament and Multi-ligament Knee Injuries

**DOI:** 10.21203/rs.3.rs-2488145/v1

**Published:** 2023-01-20

**Authors:** Cale A. Jacobs, Robert C. Schenck, Leorrie A. Watson, Caitlin E. W. Conley, Darren L. Johnson, Austin V. Stone, Christian Lattermann, Dustin L. Richter

**Affiliations:** Massachusetts General Brigham Sports Medicine; University of New Mexico; University of New Mexico; University of Kentucky; University of Kentucky; University of Kentucky; Massachusetts General Brigham Sports Medicine; University of New Mexico

**Keywords:** inflammation, cytokine, anterior cruciate ligament, multi-ligament knee injury

## Abstract

**Objective and design:**

The purpose of this study was to compare synovial concentrations of pro- and anti-inflammatory cytokines between multiple-ligament knee injured (MLKI) and anterior cruciate ligament (ACL)-injured patients.

**Subjects:**

14 patients with MLKI and 10 patients with isolated ACL injury

**Methods:**

Synovial fluid was aspirated from the operative knee at the time of surgery and the concentrations of pro- and anti-inflammatory markers in the synovial fluid were determined. Structures injured, the time between injury and surgery, and demographic factors were collected. Linear regressions were used to determine the association between injury pattern and synovial inflammatory markers when controlling for age, BMI, and time between injury and surgery.

**Results:**

When adjusting for group differences in age, BMI and the time between injury and surgery, no group differences were detected (interferon gamma (p = 0.11), interleukin-1beta (IL-1b, p = 0.35), IL-2 (p = 0.28), IL-4 (p = 0.64), IL-6 (p = 0.37), IL-8 (p = 0.54), IL-10 (p = 0.25), IL-12p70 (p = 0.81), IL-13 (p = 0.31), and tumor necrosis factor-alpha (p = 0.90)).

**Conclusion:**

Synovial fluid inflammatory markers did not differ between MLKI and isolated ACL injuries. MLKIs have a greater prevalence of postoperative arthrofibrosis when compared to isolated ACL injuries; however, this may be due in part to factors other than the inflammatory status of the joint.

## Introduction

Arthrofibrosis, a fibrotic joint disorder characterized by excessive collagen production and adhesions, is a known potential complication after knee surgery and often results in pain and restricted range of motion. [27] Pro-inflammatory cytokines are involved in the development of arthrofibrosis by inducing proliferation and differentiation of fibroblasts to myofibroblasts.[3] Myofibroblasts are contractile and apoptosis of myofibroblasts is necessary for wound healing. In the absence of apoptosis of myofibroblasts, the joint may experience a build-up of contractile fibrotic scar tissue thereby limiting mobility.[16, 25, 27] As such, pro-inflammatory cytokines and T-cells are thought to mediate the pathogenesis of arthrofibrosis.[1, 16, 18, 19, 25, 27]

The prevalence of postoperative arthrofibrosis is greater following surgical treatment of multi-ligament knee injury (MLKI) than with isolated anterior cruciate ligament (ACL) injuries.[12, 13, 17] It has been proposed that the increased prevalence of postoperative arthrofibrosis after MLKIs compared to isolated ACL injuries may be due to increased inflammation secondary to greater trauma to the joint.[9, 20] The purpose of this study was to compare synovial concentrations of pro- and anti-inflammatory cytokines between patients that have a MLKI or isolated ACL injury. We hypothesized that MLKI would demonstrate a more pro-inflammatory state than those with an isolated ACL injury at the time of reconstruction.

## Materials And Methods

### MLKI Patients

This was an ancillary study performed in conjunction with the Surgical Timing and Rehabilitation Trial for Multiple Ligament Knee Injuries (STaR Trial; NCT03543098). The current study shared the same inclusion and exclusion criteria as the STaR Trial but was treated as a separate protocol. Eligible patients were first approached regarding participation in the STaR Trial; however, participation in the current study was not dependent on STaR Trial participation. Those that opted not to take part in the STaR Trial could participate in the current observational study. This was an exploratory analysis to be used for future hypothesis generation and as such, an a priori sample size calculation was not performed.

Fifteen patients with a clinical and MRI-confirmed MLKI consented to participate in this IRB-approved prospective multi-center study. Participants were recruited between November, 2019 and March, 2021 from two academic medical centers with both being the only Level 1 trauma center in their respective regions. Males and females between the ages of 16 and 55 with a MLKI (defined as a complete grade III injury of two or more ligaments) were eligible to participate. Individuals with a nerve injury or biceps or popliteus tendon rupture/avulsion were not excluded. The following exclusion criteria were employed: a history of prior knee ligament surgery of the involved knee, a torn or avulsed patellar or quadriceps tendon, any condition that precluded early post-surgical weight bearing or range of motion exercise within 7 days of surgery (such as a periarticular or long bone fracture, a skin or soft tissue injury, surgery for extensor mechanism rupture or avulsion, or vascular graft surgery), a condition that required use of an external fixator to maintain reduction of the knee or soft tissue/open wound management for greater than 10 days, were unable to weight bear on the contralateral uninjured leg, a traumatic brain injury that limited their ability to participate in their postoperative care or any other condition that would preclude the ability to comply with postoperative guidelines.

#### ACL Patients:

Samples collected from the MLKI patients were compared to synovial fluid samples collected from 10 patients with isolated ACL injuries that were in the placebo control group from a previous IRB-approved randomized controlled trial (NCT01692756). Patients were included if they met the following criteria: clinical confirmation of an isolated ACL injury (positive Lachman test with intraarticular effusion, normal varus-valgus test), between the ages of 14 and 32, had an isolated ACL injury (no more than a grade 1 MCL injury defined clinically), no history of previous ACL injury (ipsilateral or contralateral knee), injury that occurred during performance of a sporting activity, and growth plates that were closed as documented by plain x-ray. Patients were excluded if they had a history of any inflammatory disease, immune compromise, chronic use of NSAIDs, history of intra-articular cortisone injection into either knee within the prior three months, or prior knee surgery. Patients with a posterior cruciate ligament injury or more than grade I injuries to either collateral ligament were excluded; however, patients were not excluded based on meniscal status.

### Data Collection and Analysis

Details of the clinical knee dislocation classification (KD grade),[21] the time between injury and surgery, and demographic factors (age, sex, BMI) were collected. The KD classification criteria are presented in Table 1. Joint aspiration was performed by the treating surgeon in the operating room after the knee had been prepped and draped aseptically through a superolateral parapatellar approach. Aspirated synovial fluid was spun at 3500 RPM for 10 minutes and the supernatant was frozen at −80°C. Commercially available ELISA kits (Proinflammatory Panel 1, Meso Scale Diagnostics) were used to determine concentrations of pro- and anti-inflammatory markers in the synovial fluid including interferon gamma (IFN-γ), interleukin-1beta (IL-1b), IL-2, IL-4, IL-6, IL-8, IL-10, IL-12p70, IL-13, and tumor necrosis factor alpha (TNF-a). Assays were completed per the manufacturer’s instructions and were run in duplicate. For all plates, intra-assay coefficients of variance were < 9.5. For values that were below the lower limit of detection, ½ LLOD (lowest level of detection) were reported for statistical purposes. To avoid any potential bias, samples were analyzed by the biomarker assessment core laboratory at one of the participating universities.

### Statistical Analyses

None of the variables assessed were normally distributed, and as such, synovial biomarkers, injury and patient factors were compared between patients with a MLKI or ACL injury using Mann-Whitney U tests. To account for potential group differences in age, BMI, and the time since injury, biomarker values were log transformed to allow linear regression analyses to determine the association between injury pattern and synovial inflammatory markers when controlling for these patient and injury characteristics. Pearson Product Moment correlations were used to assess the associations between log transformed biomarker concentrations and age, BMI, and time between injury and surgery independent of injury type. Finally, within the MLKI group, we compared synovial biomarker concentrations between KD I and KD III injuries. Due to the small sample size for this exploratory subgroup analysis, we did not perform statistical tests comparing the groups. Rather, effect sizes were calculated to aid in interpretation of this exploratory subgroup analysis. Effect sizes for two-sample nonparametric data were calculated by dividing the Z score by the square root of the sample size. Effect sizes < 0.30 are considered small, 0.30 and 0.49 as average, and ≥ 0.50 considered large.[8] All analyses were performed using SPSS Statistics 28 (IBM, Armonk, NY).

## Results

Synovial fluid was successfully aspirated from the injured joint at the time of surgery for 14/15 MLKI patients and all 10 ACL patients (Table 2). Of the patients with a MLKI, 9 were KD I, three were KD IIIM (medial) and 2 were KD IIIL (lateral). The MLKI group was significantly older with a greater BMI than the ACL group (Table 2). Unadjusted biomarker concentrations did not differ between groups, nor were group differences noted when controlling for age, BMI, and time between injury and reconstructive surgery (p-values ranging from 0.11 to 0.90; Table 3). For the most part, time from injury, age, and BMI were not independently associated with synovial inflammatory markers with the one exception of age being significantly associated with IL-2 (Table 4). In the exploratory comparison between injury patterns, 8/14 (57.1%) patients were reconstructed within 3 months of injury, 3/14 (21.4%) were reconstructed between 3 and 6 months after injury, and 3/14 (21.4%) were chronic injuries that were treated more than 6 months after injury (Table 5). There were no marked differences in biomarker concentrations between KD I and KD III injuries as no large effect sizes were noted (Table 6).

## Discussion

Contrary to our hypothesis, synovial markers of inflammation did not differ between patients with a MLKI or isolated ACL injury. Patients with a MLKI were significantly older with greater BMIs, and there tended to be greater time between injury and surgery (p = 0.11) than the isolated ACL group. Because increased age, BMI, and time between injury and surgery have been associated with reduced risk of arthrofibrosis,[9, 14, 17, 20] these factors were statistically controlled for in our analyses. However, by and large these three factors were not independently associated with synovial inflammatory markers at the time of surgery (Table 3). Historically, operating too soon after ACL injury was thought to increase the risk of arthrofibrosis secondary to the joint being acutely inflamed.[22, 23] However, over the past 25 years, there has been a gradual shift to ACL reconstruction earlier after injury as multiple studies and systematic reviews have demonstrated that earlier ACL reconstruction does not increase the risk of arthrofibrosis.[7, 13, 26] The lack of difference in inflammatory profiles between MLKI and ACL injuries in the current pilot study then raises the provocative question of whether earlier surgery might also be a safe option for patients with a MLKI.

Just as intriguing as the lack of difference in inflammatory markers between MLKI and isolated ACL injuries was that the exploratory subgroup results also suggest that there may not be dramatic differences in the intraarticular inflammatory profile between KD I and KD III injuries. If the severity of injury is not associated with a greater cytokine burden at the time of surgery, then the increased prevalence of arthrofibrosis after MLKI may be due, at least in part, to factors other than the inflammatory status of the joint. It is unknown whether the postoperative inflammatory burden secondary to the surgical insult and/or patient demographic or socioeconomic factors that may alter access to or willingness to comply with physical therapy may influence the increased prevalence of arthrofibrosis after MLKI.

In the current study, the synovial fluid concentrations of inflammatory markers did not differ between patients with an isolated ACL or MLKI patients at the time of surgery. However, the surgical insult of ACL reconstruction reinitiates the inflammatory process with synovial fluid concentrations of proinflammatory cytokines increasing sharply 1 week after surgery. While cytokine concentrations lessen by 4 weeks after ACL reconstruction, they remain greater than what was observed within 10 days of the initial injury. It is unclear if this represents the joint’s response to a second large inflammatory event over a relatively short period of time, or if the surgical insult itself is more traumatic than the initial injury. If the latter is true, then perhaps the additional surgical procedures associated with MLKI might result in increased postoperative inflammation and cytokine burden relative to isolated ACL reconstruction, which could contribute to the greater prevalence of arthrofibrosis after MKLI.

Both pre- and postoperative rehabilitation may contribute to the increased prevalence of arthrofibrosis after MLKI as well. Preoperatively, limited range of motion, effusion, and swelling have been previously reported to be associated with arthrofibrosis following ACL reconstruction.[5, 16, 22] It is important to note that the volume of swelling in the joint is not synonymous with increased cytokine concentrations, as the volume aspirated from the joint was previously reported to not differ between ACL patients with normal versus dysregulated inflammatory responses to injury.[10] Mechanistically, increased cytokine activity may increase fibrosis thereby resulting in arthrofibrosis whereas the volume of swelling may physically hinder the ability to fully flex or extend the knee. Additionally, a joint effusion may result in arthrogenic inhibition of the quadriceps limiting active extension. Similarly, while postoperative rehabilitation protocols after MLKI will differ based on the structures involved and procedures performed, one of the primary goals of the early postoperative rehabilitation is to restore knee range of motion and activation of the quadriceps without over-stressing the involved tissues.[15] Until recently, many rehabilitation protocols advocated immobilization of the knee after surgery for MLKI for anywhere from 1 to 6 weeks;[11] however, early initiation of range of motion has led to better range of motion outcomes without negatively affecting stability.[17] As such, reducing effusion and safely restoring range of motion and activating the quadriceps both prior to and following surgery are imperative to reduce the risk of arthrofibrosis no matter if treating a single or multiple ligament injury.

Patient factors may also influence the increased prevalence of arthrofibrosis after MLKI. In the current study, patients with a MLKI were older with increased BMI. While both of these factors may potentially lessen the risk of arthrofibrosis,[9, 14] other differences between MLKI and ACL patient populations may be involved. The majority of MLKIs are the result of traumatic mechanisms such as motor vehicle accidents rather than sports injuries.[2, 20] Among other factors, there are differences in socioeconomic status, level of education, and insurance provider between orthopaedic trauma and sports medicine patients, with orthopaedic trauma patients also being more often male.[4, 24] Male sex, lower education, low socioeconomic status, and public insurance are associated with reduced physical therapy utilization.[6, 28] Population differences that impact access to and/or the ability to comply with postoperative rehabilitation may influence the increased prevalence of arthrofibrosis after MLKI compared to isolated ACL injury.

### Limitations

The results of this pilot study should be used for hypothesis generation. The sample size was too small to assess the relationship between inflammatory markers and arthrofibrosis. We attempted to account for potential confounders and analyzed the synovial fluid data using both nonparametric methods as well as with regressions to statistically account for group differences in age, BMI, and time between injury and surgery. However, there are statistical limitations of including 3 confounding variables in our regression analyses with a relatively small sample size. Larger studies are necessary to mitigate these limitations and further elucidate the biological, rehabilitation, and patient-related factors that may contribute to the increased risk of arthrofibrosis following MLKI.

## Conclusions

Contrary to our hypothesis, synovial markers of inflammation did not differ between MLKI and isolated ACL injuries. It has been well established that MLKI have a greater prevalence of postoperative arthrofibrosis when compared to isolated ACLs; however, this may be due in part to factors other than the inflammatory status of the joint. The lack of difference in inflammatory profiles between MLKI and ACL injuries raises the question of whether earlier surgery might be a safe option for MLKI. However, the biological and/or patient-related factors that contribute to increased arthrofibrosis after MLKI have yet to be elucidated. Future research will include examination of these markers, analysis of arthrofibrotic knees, and the etiologies of the stiff knee joint.

## Figures and Tables

**Table 1:** Knee Dislocation (KD) Classification Criteria[21]

KD Classification	Description
KD I	Disruption of one cruciate ligament (ACL or PCL) and one or both collaterals (LCL or MCL)
KD II	Disruption of ACL and PCL; no injury to either collateral
KD IIIL	Disruption of ACL, PCL, and LCL or posterolateral corner
KD IIIM	Disruption of ACL, PCL, and MCL or posteromedial corner
KD VI	Disruption of ACL, PCL, LCL, and MCL or both corners
KD V	Fracture-dislocation

**Table 2:** Comparison of patient factors (median (interquartile range)) between those with multiple ligament knee injuries (MLKI) versus isolated anterior cruciate ligament (ACL) injury.

Variable	MLKI	ACL	p-value	Effect Size*
N	14	10	-	-
Age	32.7 (23.5–45.1)	18.2 (15.6–20.3)	**<0.001**	**0.71**
Sex (F/M)	4/10	6/4	0.21	-
BMI	29.5 (27.1–36.3)	23.1 (20.2–24.6)	**<0.001**	**0.65**
Days since injury	106 (23–199)	28 (25–40)	0.11	0.33

* Nonparametric effect sizes: < 0.30 are considered small, 0.30 and 0.49 as average, and ≥ 0.50 considered large

**Table 3:** Comparison of synovial fluid inflammatory markers (mean ± standard deviation) between those with MLKI versus isolated ACL injury.

Variable	MLKI	ACL	P-value	Effect Size*	Adjusted P†
N	14	10	-	-	-
IFNg	1.20 (0.50–3.31)	2.81 (1.65–6.05)	0.07	0.37	0.11
IL-1β	0.06 (0.03–0.14)	0.20 (0.05–0.47)	0.11	0.34	0.35
IL-2	0.28 (0.05–0.38)	0.38 (0.26–0.87)	0.10	0.35	0.28
IL-4	0.03 (0.01–0.08)	0.02 (0.01–0.09)	> 0.99	0.00	0.64
IL-6	3.0 (1.0-143.8)	15.7 (4.3–66.0)	0.37	0.19	0.37
IL-8	30.8 (13.8–94.0)	25.5 (14.2–54.5)	0.98	0.01	0.54
IL-10	0.16 (0.06–0.62)	0.28 (0.19–0.43)	0.26	0.24	0.25
IL-12p70	0.13 (0.06–0.37)	0.14 (0.09–0.37)	0.71	0.08	0.81
IL-13	0.28 (0.12–1.29)	0.70 (0.33–1.42)	0.15	0.30	0.31
TNFa	1.74 (1.17–2.60)	1.38 (1.20–1.94)	0.71	0.08	0.90

* Nonparametric effect sizes: < 0.30 are considered small, 0.30 and 0.49 as average, and ≥ 0.50 considered large

† Adjusted P-values are the results of multiple variable linear regression controlling for age, BMI, and time since injury

**Table 4:** Correlations (r value (p-value) between synovial inflammatory markers and age, BMI, and time from injury.

Variable	Age	BMI	Days Since Injury
IFNg	−0.27 (p = 0.21)	−0.07 (p = 0.76)	0.13 (p = 0.54)
IL-1β	−0.35 (p = 0.10)	−0.21 (p = 0.32)	0.01 (p = 0.95)
IL-2	−0.42 (p = 0.04)	−0.24 (p = 0.26)	0.02 (p = 0.93)
IL-4	−0.03 (p = 0.90)	0.10 (p = 0.65)	0.14 (p = 0.51)
IL-6	−0.16 (p = 0.47)	0.14 (p = 0.52)	0.07 (p = 0.73)
IL-8	−0.02 (p = 0.92)	0.35 (p = 0.10)	−0.10 (p = 0.65)
IL-10	−0.25 (p = 0.24)	0.09 (p = 0.69)	−0.11 (p = 0.60)
IL-12p70	−0.15 (p = 0.47)	−0.03 (p = 0.88)	0.03 (p = 0.88)
IL-13	−0.23 (p = 0.28)	−0.07 (p = 0.76)	0.03 (p = 0.91)
TNFa	0.05 (p = 0.83)	0.33 (p = 0.12)	0.08 (p = 0.72)

**Table 5:** Time between injury and surgery for those with KD I and KD III injuries.

Time between injury and surgery	KD I (N = 9)	KD III (N = 5)
< 6 weeks	4 (44.4%)	2 (40%)
6 to 12 weeks	1 (11.1%)	1 (20%)
3 to 6 months	2 (22.2%)	1 (20%)
6 to 12 months	1 (11.1%)	0 (0%)
> 12 months	1 (11.1%)	1 (20%)

**Table 6:** Synovial fluid inflammatory markers (mean ± standard deviation) between those with KD I vs. KD III MLKI.

Variable	KD I	KD III	Effect Size*
N	9	5	-
IFNg	1.26 (0.99–2.90)	0.31 (0.21–3.34)	0.30
IL-1β	0.09 (0.02–0.15)	0.03 (0.03–0.11)	0.14
IL-2	0.28 (0.14–0.43)	0.05 (0.05–0.37)	0.25
IL-4	0.04 (0.01–0.08)	0.02 (0.01–0.07)	0.27
IL-6	4.38 (1.25–156.56)	1.66 (0.66–200.77)	0.12
IL-8	30.76 (13.25–74.03)	30.99 (9.77–179.65)	0.05
IL-10	0.16 (0.06–0.64)	0.17 (0.06–0.78)	0.05
IL-12p70	0.18 (0.07–0.38)	0.08 (0.03–1.06)	0.20
IL-13	0.28 (0.18–0.94)	0.28 (0.12–2.19)	0.07
TNFa	1.97 (1.14–2.46)	1.31 (0.84–2.80)	0.05

* Nonparametric effect sizes: < 0.30 are considered small, 0.30 and 0.49 as average, and ≥ 0.50 considered large
